# Novel Time-Delay Side-Collision Warning Model at Non-Signalized Intersections Based on Vehicle-to-Infrastructure Communication

**DOI:** 10.3390/ijerph18041520

**Published:** 2021-02-05

**Authors:** Nengchao Lyu, Jiaqiang Wen, Chaozhong Wu

**Affiliations:** 1 Intelligent Transportation Systems Research Center, Wuhan University of Technology, Wuhan 430063, China; lnc@whut.edu.cn (N.L.); wenjq@whut.edu.cn (J.W.); 2 National Engineering Research Center for Water Transport Safety, Wuhan 430063, China

**Keywords:** non-signalized intersections, vehicle-to-infrastructure communication, time-delay, collision warning, simulated driving

## Abstract

In complex traffic environments, collision warning systems that rely only on in-vehicle sensors are limited in accuracy and range. Vehicle-to-infrastructure (V2I) communication systems, however, offer more robust information exchange, and thus, warnings. In this study, V2I was used to analyze side-collision warning models at non-signalized intersections: A novel time-delay side-collision warning model was developed according to the motion compensation principle. This novel time-delay model was compared with and verified against a traditional side-collision warning model. Using a V2I-oriented simulated driving platform, three vehicle-vehicle collision scenarios were designed at non-signalized intersections. Twenty participants were recruited to conduct simulated driving experiments to test and verify the performance of each collision warning model. The results showed that compared with no warning system, both side-collision warning models reduced the proportion of vehicle collisions. In terms of efficacy, the traditional model generated an effective warning in 84.2% of cases, while the novel time-delay model generated an effective warning in 90.2%. In terms of response time and conflict time difference, the traditional model gave a longer response time of 0.91 s (that of the time-delay model is 0.78 s), but the time-delay model reduced the driving risk with a larger conflict time difference. Based on an analysis of driver gaze change post-warning, the statistical results showed that the proportion of effective gaze changes reached 84.3%. Based on subjective evaluations, drivers reported a higher degree of acceptance of the time-delay model. Therefore, the time-delay side-collision warning model for non-signalized intersections proposed herein can improve the applicability and efficacy of warning systems in such complex traffic environments and provide reference for safety applications in V2I systems.

## 1. Introduction

Intersections are critical components of roadways, but they bear the greatest safety risk among traffic environments [1]. At intersections, there are complicated traffic flows that can create conflicts between turning and straight-trajectory vehicles, which significantly impairs intersection safety [2]. At non-signalized intersections in particular, the lack of control measures, such as time separation and space isolation, leads to a higher frequency of collisions than at signalized intersections. Therefore, it is of great significance to develop collision warning systems for non-signalized intersections that accurately predict conflict and warn drivers, thereby reducing collisions caused by driver miscalculation of risk and thus improving safety.

Due to limitations in sensor performance, collision warning systems that rely only on in-vehicle sensors are limited in accuracy and range, which makes them ill-equipped for complex traffic environments such as intersections, particularly non-signalized ones [3]. Current collision warning systems mainly use various sensors installed on the vehicle to perceive the surrounding information. Their main application is the forward collision warning system, which essentially measures the collision risk between the host vehicle and the vehicle ahead in the same lane. However, due to the limited sensing range of the sensor, the on-board collision warning system cannot accurately identify the risk relationship between the vehicle and the side-oncoming vehicle [4]. Therefore, a side-collision warning system has not yet been extensively verified in real traffic. Especially at non-signalized intersections with complex conditions, for vehicles heading to the intersection area, the on-board sensors may have a smaller perception angle or be blocked by large vehicles, roadside buildings, roadside trees, and road ancillary facilities [5]. In this case, the vehicles at the near-end cross entrance cannot be identified, which is likely to cause side-collision accidents. For non-signalized intersections, the traffic environment is more complicated than that of road sections where no traffic converges or separates. The information used for risk identification and side-collision warning needs to be more complete and more accurate [6]. Vehicle-to-infrastructure (V2I) communication systems, however, offer more robust information exchange, and thus, warnings. Therefore, the target information can be acquired by the roadside fixed detection device, and V2I is used to realize the interaction between roadside perception information and on-board perception information. As a result, it enhances the vehicle’s perception of target information, and improves the accuracy of the collision warning system for identifying the risk of side-collision at non-signalized intersections. Li et al. developed a method to fuse in-vehicle and roadside information, providing the foundation for collision warnings [7]. Zardosht et al. developed a model that infused information on surrounding vehicles using V2I technology and analyzed collision risks through a decision module [8]. In a V2I environment, Zhao et al. found that in-vehicle warnings positively affect driving behavior [9]. Therefore, it has become a justified research endeavor to study the collision warning system at non-signalized intersections by using the information interaction capabilities provided by the V2I technology.

V2I communication technology has been widely practiced in the study of traffic safety at intersections [10]. Analysis of intersection accidents shows that, in most cases, drivers ignore or see other road users with priority rights of way too late and they cannot react in time. Appropriate warning information supports the driver’s attention distribution and driving behavior, and can be helpful in improving the driver’s reaction [4]. Studies have confirmed that vehicles equipped with intersection assistance systems can reduce the number of collisions and injuries [11]. Intersection assistance systems have been recognized as one of the main countermeasures to reduce the collision at intersections. Warning drivers through visual and auditory forms [6], the intersection assistance system can help drivers perceive potential dangers in advance [12], adopt safer braking strategies, and effectively reduce the frequency and severity of collisions [13,14]. It is very useful for vulnerable line-of-sight and/or traffic-violation-prone environment to build a cooperative intersection collision warning system by using V2I communication [5]. Therefore, based on the theories of time slot and space reservation, the main technologies and solutions for cooperative work at non-signalized intersections have been promoted [15]; particularly, the collision warning model at non-signalized intersections has been researched.

At present, time-to-collision (TTC) [16], post-encroachment time (PET) [17], and other relevant parameters are used as cross-conflict indicators in collision warning models [18,19]; at intersections in particular, spatial proximity is commonly used. Alongside relevant parameters, V2I has been introduced in the development and deployment of collision warning models for non-signalized intersections. Liu et al. divided an intersection into small area grids with a resolution of n × m and used the degree of grid overlap occupied by the physical form of the vehicle at a particular moment to determine whether collisions would occur between vehicles [20]. Huang et al. distinguished vehicle collision type according to the lateral offset and used the time to the closest point approach model and the separating axis theorem to detect cross collisions [21]. Liu et al. selected the circular area where the vehicle was located as the detection range and calculated the conflict risk between vehicles using the direct distance and speed vector of the circular areas [22]. Relevant research has also been carried out to describe the conflict risk at intersections according to the approach degree of vehicles in the time dimension. Wang et al. established the accident probability calculation algorithm according to the time of vehicles passing through the conflict zone [23]. Li et al. selected a highway main road and entrance ramp as a testbed and proposed an extended TTC risk estimation model based on the angle between the driving direction and the connecting line of vehicles [24]. Ma et al. considered vehicle size to construct a conflict recognition model of the confluence area based on a PET algorithm [25]. According to the time-window method, Wang et al. dynamically predicted the time-varying distance between a host vehicle and a remote vehicle at intersections and judged the collision risk [26]. However, existing research is mainly based on the motion state of vehicles at a certain time to extract spatial- or temporal-related indicators and then identify potential conflicts between vehicles. For the existing side-collision warning model at non-intersections, neither the movement of the vehicle from the warning information triggering to the driver’s action, nor the movement of the vehicle during deceleration episodes, are considered. The driver’s response characteristics and braking characteristics during normal driving are ignored.

**Figure 1:**
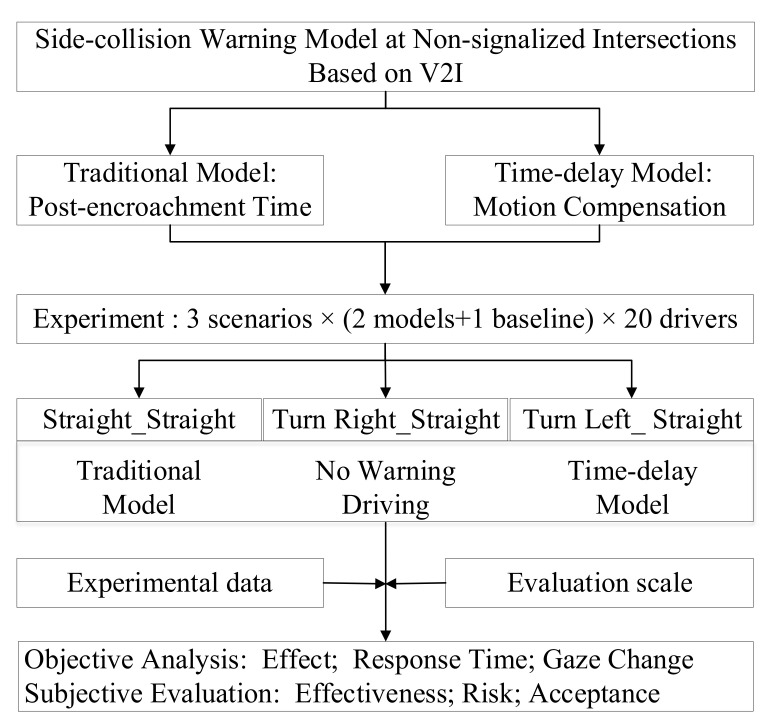
The overall framework of this study. V2I (Vehicle-to-Infrastructure) refers to the communication between the vehicle and the infrastructure.

To bridge this gap, in the V2I environment, a side-collision warning model based on time-delay at non-signalized intersection is proposed, and its demonstration and analysis are carried out. The overall framework of this study is shown in Figure 1. Firstly, the traditional collision warning model is summarized, and a time-delay side-collision warning model is established according to the principle of motion compensation. Secondly, three experimental scenarios were constructed, and three driving conditions were designed: the traditional side-collision warning model, the time-delay side-collision warning model, and the baseline (driving without a warning model). Drivers were recruited to carry out driving simulation experiments, and the corresponding data and evaluation scale were collected. Finally, from the perspectives of objective analysis and subjective evaluation, the effects of the proposed side-collision warning model and the traditional side-collision warning model are compared.

The purpose of this study was to verify the safety performance of the side-collision warning model at non-signalized intersections. The rest of the study is organized as follows: Section 2 introduces the side-collision warning model for non-signalized intersections in detail, including the traditional side-collision warning model and the time-delay side-collision warning model; Section 3 describes the experimental platform based on V2I and experimental design scheme; Section 4 presents the analysis of experimental results; and Section 5 is the conclusion part of this paper.

## 2. Side-Collision Warning Model for Intersections

In the V2I environment, with the combined advantages of on-board perception and roadside perception, the vehicle can obtain more and richer information; even moving target information that is not within the driver’s field of vision can be captured. This information provides reliable data support for the research and verification of collision warning models at non-signalized intersections. Combined with the existing research, the principle and limitation of the traditional side-collision warning model at non-signalized intersections is summarized and explained. On this basis, further considering the driver’s response characteristics and system braking characteristics and other detailed factors, a time-delay side-collision warning model was constructed.

### 2.1. Model 1: Traditional Side-Collision Warning Model for Non-Signalized Intersections

**Figure 2:**
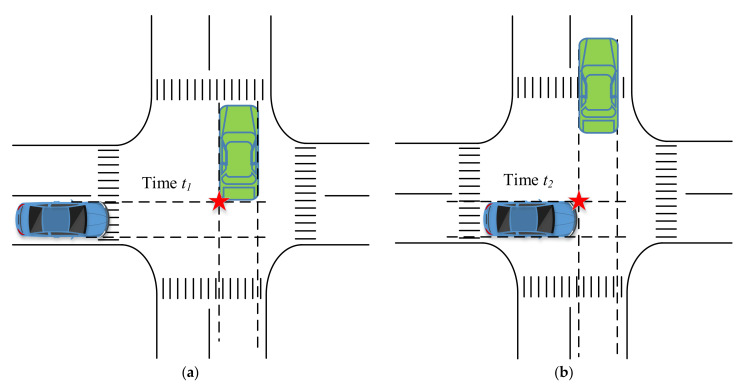
The concept of traffic conflict indicator post-encroachment time (PET). (**a**) First arriving vehicle leaves. (**b**) Later arriving vehicle enters.

According to existing research, with the traditional warning model, it is usually assumed that the host and remote vehicles’ state information at a particular moment remains unchanged, and on this basis, the subsequent risk identification and warning decision is completed. In this study, PET was selected as a collision risk indicator, and the threshold was set to be 1.5 s (according to the literature) [27]. For vehicles with spatial conflicts, PET is the time interval between when the first vehicle leaves the conflict point and when the second vehicle enters the conflict point, as shown in Figure 2.

Basic motion information was extracted from the host vehicle (HV) and remote vehicle (RV) at a certain starting moment, and the subsequent motion state control model of the vehicle was constructed as follows:

(1) $$S={\left[{\left(x_{t_{i}}-a_{0}\right)}^{2}+{\left(y_{t_{i}}-b_{0}\right)}^{2}+{\left(z_{t_{i}}-c_{0}\right)}^{2}\right]}^{1/2}{|}_{t_{i}=t_{0}}$$

(2) $$v=v_{t_{i}}{|}_{t_{i}=t_{0}}$$

In the formula, $\left(x_{t_{i}},y_{t_{i}},z_{t_{i}}\right)$ represents the position information of the vehicle at a certain moment during the driving process, $t_{i}$ represents a certain moment when the vehicle was driving towards the intersection, and $v_{t_{i}}$ represents the driving speed of the vehicle at the moment $t_{i}$. $\left(a_{0},b_{0},c_{0}\right)$ represents the fixed geometric position parameter of the intersection, and $t_{0}$ represents the starting moment of the conflict risk analysis between the HV and RV.

By calculating the characteristic indices of the HV and RV, the prediction model of collision risk between HV and RV was established:

(1). When the HV reached the conflict point first and the RV reached the conflict point second:
  (3) $$t_{1}=\frac{S_{h}+L_{h}+W_{r}}{V_{h}},t_{2}=\frac{S_{r}}{V_{r}}\left(t_{2}>t_{1}\right)$$
(2). When the RV reached the conflict point first and the HV reached the conflict point second:
  (4) $$t_{1}=\frac{S_{r}+L_{r}+W_{h}}{V_{r}},t_{2}=\frac{S_{h}}{V_{h}}\left(t_{2}>t_{1}\right)$$
  (5) $$PET=t_{2}-t_{1}$$

In the formula, $t_{1}$ represents the time when the first arriving vehicle left the conflict point, and $t_{2}$ represents the time when the later arriving vehicle reached the conflict point; $S_{h}\left(S_{r}\right)$ represents the distance between the HV (RV) and the conflict point; $L_{h}$ and $L_{r}$ represent the length of the HV and RV, respectively; $W_{h}$ and $W_{r}$ represent the width of the HV and RV, respectively; and $V_{h}$ and $V_{r}$ represent the speed of the HV and RV, respectively.

### 2.2. Model 2: Time-Delay Side-Collision Warning Model for Non-Signalized Intersections

**Figure 3:**
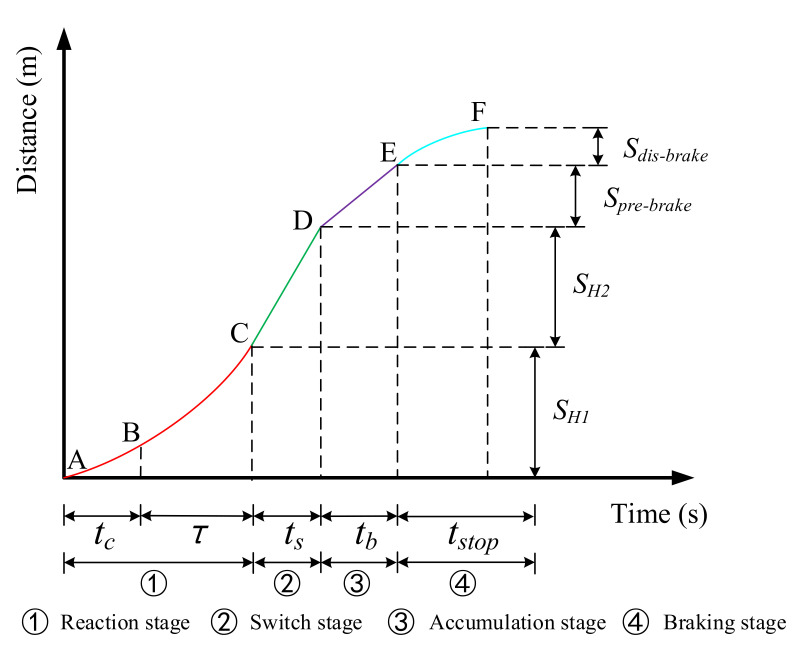
Analysis of vehicle movement stages.

For traditional side-collision warning models at non-signalized intersections, there are usually the following assumptions: (1) the HV drives to the intersection at a constant speed; and (2) the driving direction of HV will not change. These assumptions ignore the driver’s operation process of the HV, and also ignore the process of vehicle movement posture transformation, especially the movement change from the successful triggering of the warning to the beginning of the braking effect. This model assumption may not be appropriate in the real traffic environment, and has a great impact on the accuracy and timeliness of the warning model. Therefore, based on the influence of the driver and system characteristics, the driver’s reaction, judgment, and execution process, the braking effect accumulation process and the vehicle’s position update after driver’s operation are considered. It plays an important role in improving the performance of side-collision warning models at non-signalized intersections. In this study, a motion compensation method was used to predict the future motion state information of HV, and the collision risk identification and warning decision are completed based on this.

(1). Preliminary estimation: The conflict between the HV and RV was preliminarily estimated using indicator *T*2. *T*2 refers to the time required for the later arriving vehicle to reach the conflict area when the first arriving vehicle has not yet left the conflict area [28].

(6) $$T2=\left\{\begin{array}{c} \frac{S_{r}}{V_{r}},\frac{S_{h}}{V_{h}}<\frac{S_{r}}{V_{r}}<\frac{S_{h}+L_{h}+W_{r}}{V_{h}}\\ \frac{S_{h}}{V_{h}},\frac{S_{r}}{V_{r}}<\frac{S_{h}}{V_{h}}<\frac{S_{r}+L_{r}+W_{h}}{V_{r}} \end{array}\right.$$

(2). Secondary judgment: (a) Considering the vehicle’s position changes during the time period when the driver perceives the warning and system braking gradually takes effect; (b) predicting the trajectory of the vehicle after the brake takes effect; (c) judging whether the vehicle can stop completely within the allowable time or effective distance. Based on the existing emergency braking model established for the driver’s reaction process and braking process [29], this research fully analyzed the position change of the vehicle during the process from warning to complete stop, as shown in Figure 3. The movement process of the vehicle is divided into four stages, namely the reaction stage, the switching stage, the accumulation stage, and the braking stage. The movement of the vehicle in each stage has different characteristics. Point A indicates that the warning is generated, and the vehicle is approaching the intersection; point F means that the vehicle is completely stopped, and the vehicle is at a standstill; point B, point C, point D, and point E are the critical points between each stage. Moreover, the compensation distance of the HV is recorded as $S_{H1},S_{H2}$ and $S_{pre-brake}$. $S_{H1}$ represents the position change of the HV from warning generation to the reception of information by the driver; $S_{H2}$ indicates the position change of the HV during the process from releasing the accelerator to pressing the brake pedal; $S_{pre-brake}$ denotes the position change of the HV when the braking gradually takes effect. $S_{dis-brake}$. represents the position change of the vehicle from braking to a complete stop. The following will analyze the changes of vehicle movement from the warning generation to complete parking.

(7) $$S_{H}=S_{H1}+S_{H2}+S_{pre-brake}+S_{dis-brake}$$

In the formula, $S_{H}$ is the sum of the HV’s compensation and braking distance, i.e., the predicted position change.

(8) $$S_{H1}=v_{H}\left(t\right)*\left(\tau +t_{c}\right)+\frac{1}{2}a_{H}\left(t\right)*{\left(\tau +t_{c}\right)}^{2}$$

In the formula, τ and $t_{c}$ represent the driver’s cognitive reaction time and the communication delay to trigger the warning, respectively; $a_{H}\left(t\right)$ represents the acceleration of the HV at a certain moment; and $v_{H}\left(t\right)$ represents the driving speed of the HV at a certain moment. Previous studies have shown that the driver’s reaction time is between 0.5~0.85 s, so the value was set accordingly at τ=0.75 s [30]. In this study, the developed simulation driving platform based on V2I was used as the experimental environment. After many tests, it was concluded that the communication delay that triggers the warning was related to the delay of the intelligent on-board unit and human–machine interaction equipment used in the experiment. Information generation and information triggering were completed in the intelligent on-board unit and the human-machine interaction equipment, respectively; therefore, information printing was used to display the generated information and the triggered information as a visual digital display on the corresponding screen, and kept the visual numbers of both within the same lens range. At the same time, a video of the generated information and the triggered information update process was captured, and with the help of video processing software, the time difference between information generation and information triggering was calculated frame by frame (about 25 frames per second). Finally, the average value of the delay was obtained. By testing the built-in collision warning system at intersections based on V2I, it was found that the communication delay of the warning was approximately $t_{c}=0.2\;s$. This research was based on a simulation experiment platform; therefore, the measured delay values met the research requirements.

(9) $$S_{H2}=\left[v_{H}\left(t\right)+a_{H}*\left(\tau +t_{c}\right)\right]*t_{s}$$

In the formula, $t_{s}$ represents the time period, in seconds, from the driver’s release of the accelerator until the brake takes effect [31], and its value was $t_{s}=0.32\;s$.

(10) $$S_{pre-brake}=\overset{t_{b}}{\underset{0}{\int }}v\left(r\right)dr\approx \frac{1}{2}\left[v_{H}\left(t\right)+a_{H}*\left(\tau +t_{c}\right)\right]*t_{b}$$

In the formula, $t_{b}$ represents the accumulation time of the brake system, i.e., the time when the braking effect was gradually generated. The general value range was 0.3~0.75 s, so $t_{b}=0.4\;s$.

(11) $$S_{dis-brake}=v_{brake}^{2}/2a_{e}$$

In the formula, $a_{e}$ represents the deceleration during braking, and $v_{brake}$ represents the speed at the beginning of braking. The deceleration of normal vehicles during braking is generally between 4~8 m/s^2^, so $a_{e}=6\;m/s^{2}$ was selected. Therefore, the time required for the HV to stop completely was estimated:

(12) $$t_{H}=\tau +t_{c}+t_{s}+t_{b}+t_{stop}$$

In the formula, $t_{stop}$ represents the time required for the HV to complete braking.

**Figure 4:**
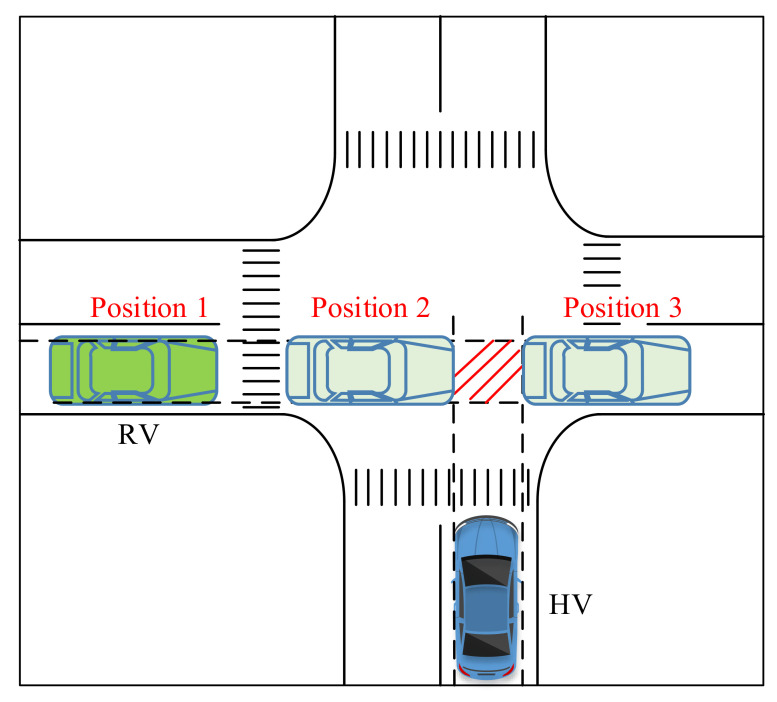
Schematic diagram of the limit position in the case of vehicle conflict.

As shown in Figure 4, if the RV entered the conflict area first, and when the RV was at position 1, the limit position 2 and the limit position 3 when the RV reached or left the conflict area was estimated by acquiring the real-time information of the RV. After calculation, the time range in which the RV was in the conflict zone was calculated as $\left(t_{r1},t_{r2}\right)$.

Through the above model, the HV’s position change and the required time was predicted. When the warning was triggered, it needed to meet the following requirements:

(13) $$\left\{\begin{array}{c} t_{H}\in \left(t_{r2}-T_{0},t_{r2}\right)\\ S_{h}\leq S_{H} \end{array}\right.$$

Among them, *T*_0_ is a fixed value that represents the proximity on the time dimension. Using surrogate safety measures (SSMs) to characterize the risk of collision is a general method for predicting the probability of a vehicles’ potential collision [32]. Generally, the risk measurement is computed by a relationship between the actual calculated value of SSMs and the critical threshold. $T_{0}$ is also a critical value attributable to the SSMs. Pre-experiments can be carried out in the traffic scenarios constructed in this experiment to complete the measurement of the $T_{0}$ value.

Similarly, if the HV entered the conflict area first, the warning triggering needed to meet the following requirements:

(14) $$\left\{\begin{array}{c} t_{H}\in \left(t_{r1}-T_{0},t_{r1}\right)\\ S_{h}\leq S_{H} \end{array}\right.$$

Based on simulated driving experiments carried out by other research groups in similar scenarios [26,33,34], the threshold $T_{0}$ was tested and the value range was given. Combined with the driver’s process of receiving and perceiving information, the value range was $T_{0}\in \left(0.6,1.2\right)$. By taking the interval of 0.1 s, different $T_{0}$ values were tested and verified, and the most appropriate value of $T_{0}$ was obtained: $T_{0}=1.0\;s$. Therefore, based on the above research, the time-delay side-collision warning model for non-signalized intersections was determined.

## 3. Simulation Experiment Design: V2I-Oriented Collision Warning System

In order to study the application effects of the two side-collision warning models at non-signalized intersections, it was planned to carry out experiments to verify the models. Considering the risks, uncontrollability, and high cost of the real vehicle test, a simulated driving experiment was adopted. Taking the driving simulator as a prototype, a V2I communication environment was constructed by establishing a communication mechanism between the traffic software and the user’s vehicle, and a simulation driving platform for V2I testing is built. Taking this simulation driving platform as the experimental environment, detailed data in each driving test were collected.

### 3.1. Experiment Platform

#### 3.1.1. Platform Composition and Functions

**Figure 5:**
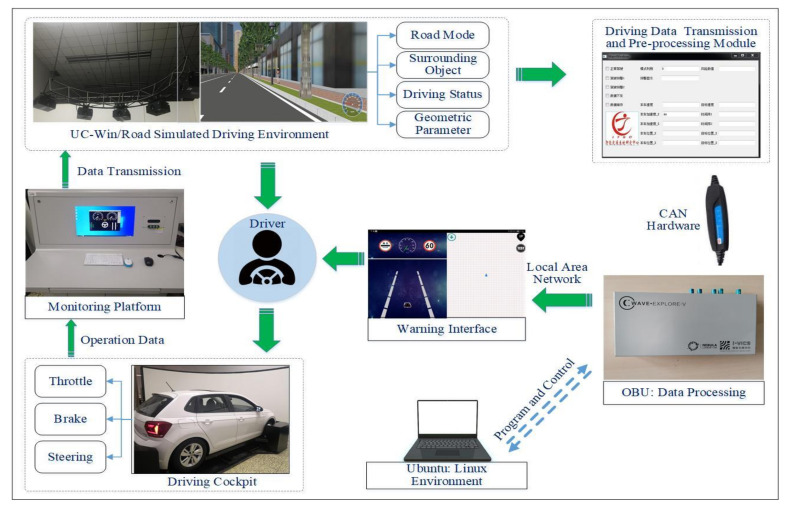
Test platform architecture of collision warning system based on V2I. UC-win/Road is a simulated driving software. CAN refers to controller area network. OBU refers to the on-board unit.

The simulation driving platform for V2I testing was based on the real vehicle (actual controller) and simulation environment (virtual object), combined with other communication interfaces, computing devices, and display devices to form a hardware-in-the-loop simulation system. The simulation driving platform was mainly composed of three parts: a driving simulator, an intelligent on-board unit (OBU) and a human–machine interface (HMI), as shown in Figure 5. The driving simulator was used to build the simulation traffic and driving environment. The OBU was mainly used to receive information, drive the model, and deliver information, and store or edit the traditional warning model and the novel time-delay warning model. The HMI was used to receive warning decisions and trigger warning information.

The platform included basic and necessary functions, such as simulation driving, V2I-based information interaction, collision risk identification, and warning. The process of each function was as follows: (1) simulation driving function: mainly composed of the driving cockpit, monitoring platform, and projection equipment. When the driver manipulated the cockpit, the operating parameters of the vehicle (throttle, brake, and steering) were uploaded to the traffic simulation environment constructed by UC-win/Road in real time via the monitoring platform, and the movement of vehicle was interpreted and reproduced in it. (2) Information interaction function: composed of data transmission and pre-processing plug-in based on software development kit (SDK), controller area network (CAN) bus transmission module and intelligent OBU. The data transmission and pre-processing plug-in could obtain the global information (road mode, surrounding targets, driving status and road geometry, etc.) in the local area of the roadside, and preprocess and encode the data. Then, the CAN bus transmission module established a communication channel with the intelligent OBU to realize the transmission of V2I information. Among them, the intelligent OBU ran under the Linux environment, and its built-in programs could be controlled by a computer under the same local area network. (3) Collision risk identification function: the intelligent OBU received the information sensed by the roadside and extracted the surrounding target information and the host vehicle information. By using the above-mentioned side-collision warning models at non-signalized intersections, the intelligent OBU could complete the collision risk identification of the host vehicle and the remote vehicle, and share the risk identification result and other basic information to the HMI through the TCP/IP communication protocol. (4) Warning function: after the HMI received the information from the intelligent OBU, it triggered the warning according to the position of the dangerous target at the non-signalized intersections.

#### 3.1.2. Human–Machine Interface

The HMI was built into a portable mobile terminal (such as a tablet or a mobile phone) and was placed on the center console of the vehicle during the experiment, such that it was always within the driver’s sight. In order to eliminate the interference of the dashboard and the HMI to the driver, the dashboard did not display any numerical information during driving, and all information was triggered and displayed by the HMI. Warning forms included an icon and alarm sound. The icon was a flashing red vehicle, and the alarm sound was a rapid “di-di” sound and voice prompts. The basic information displayed on the HMI also included the road speed limit and the real-time speed of the host vehicle.

**Figure 6:**
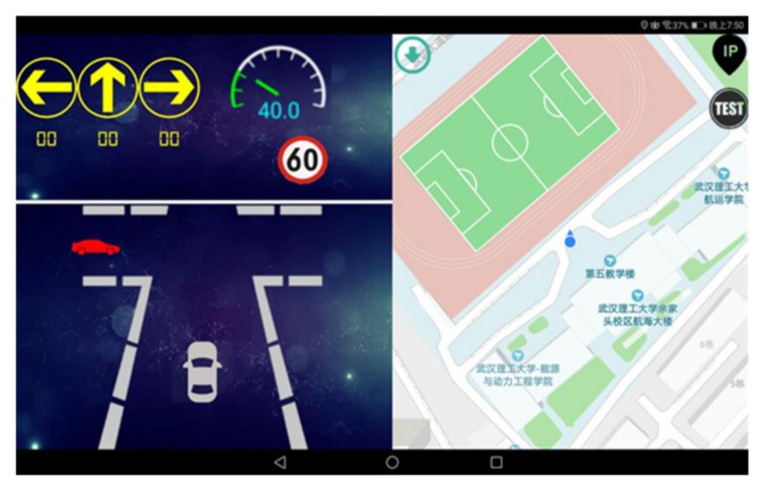
Demonstration of collision warning effect at non-signalized intersections.

As shown in Figure 6, when the host vehicle and the remote vehicle from the left approached the non-signalized intersection, the intelligent OBU judged that the host vehicle and the remote vehicle from the left had a collision risk. Immediately afterwards, on the HMI, the red flashing vehicle appeared in the corresponding dangerous area at the non-signalized intersection, accompanied by the alarm sound and the voice prompt “Attention, vehicle from left”. In this study, the traffic direction arrow did not change at the non-signalized intersection, nor did it play any role. The map on the right was not updated due to a lack of real GPS information in a simulated environment.

#### 3.1.3. System Operation Process

**Figure 7:**
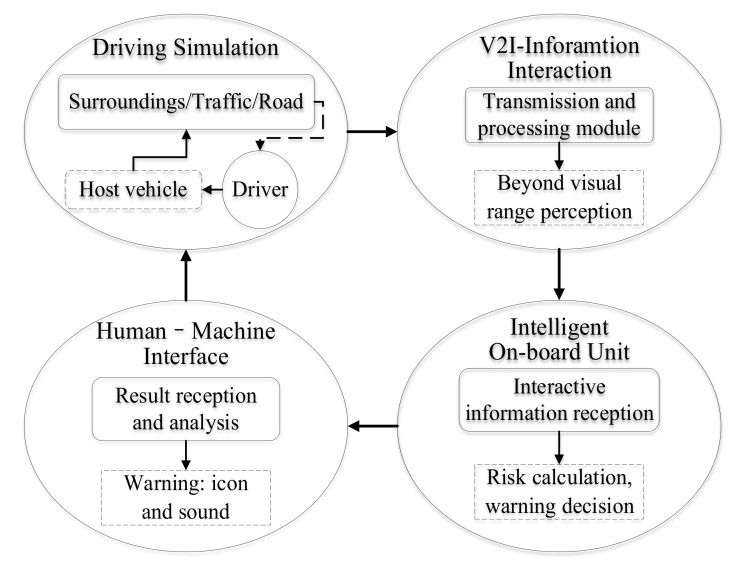
Function realization principle of warning system.

The implementation process of the collision warning system at non-signalized intersections was as follows: when the driver engaged in simulated driving, the data transmission and pre-processing module could obtain the information sensed by the roadside, and transmit it to the intelligent OBU. According to the received information, the intelligent OBU used the built-in model to complete risk calculations and warning decisions, then sent the results to the HMI. HMI triggered the corresponding information by analyzing the results to produce intervention or assistance to the driver. The realization of the entire warning system function was a closed loop, as shown in Figure 7.

Based on the simulation driving platform for V2I testing, the experimental scene was established by using UC-win/Road software (FORUM 8, Tokyo, Japan) and the joint debugging and function testing of the various equipment of the platform were completed. In this study, the HV was outfitted with on-board communication equipment, while the RV was not. The HV included a real cockpit and was operated by the test driver; the RV was generated and operated by simulation software (UC-win/Road), and it was projected on a 180° circular screen with dynamic traffic.

### 3.2. Experimental Design Instructions

#### 3.2.1. Scenario Design

**Figure 8:**
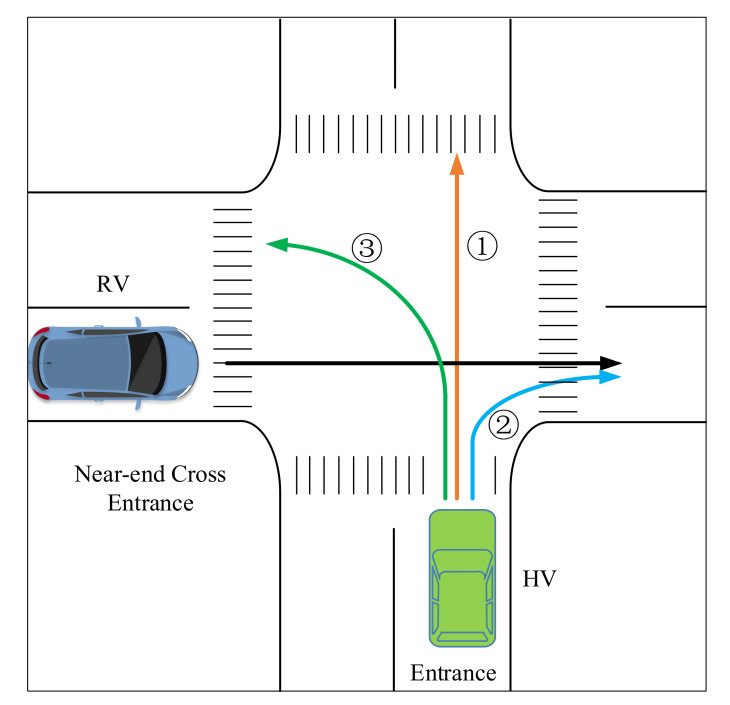
Design of vehicle conflict mode in the experimental scenarios.

Taking a two-lane non-signalized intersection as the experimental site, and according to typical dangerous situations encountered in real driving, three vehicle-to-vehicle conflict scenarios were designed on an urban road, namely, scenario 1 (HV straight–RV straight), scenario 2 (HV turn right–RV straight), and scenario 3 (HV turn left–RV straight), as shown in Figure 8. In the design of the experimental scenarios, traffic flow was generated on each road: traffic speed was 50 km/h, traffic flow was 600 pcu/h, and the vehicle types included trucks and cars. It was determined that when the vehicle was less than 150 m from the center of the intersection, it entered the risk stage of the experimental scenario, and stimulated the model to start risk calculation and decision-making. The HV was equipped with an intelligent on-board unit, with functions such as communication and calculation; the other vehicles in the simulated traffic environment were ordinary vehicles. To obtain as much warning data as possible, the risk infusion method was used in the scenarios to increase the triggering rate of conflict conditions.

#### 3.2.2. Definition of Interactive Data Type

V2I communication established a channel for the interaction between on-board perception information and roadside perception information and provided necessary information support for driving risk identification and side-collision warning at non-signalized intersections. In this study, using the interaction function based on V2I, the host vehicle obtained other information that it could not perceive. The detailed information of the remote vehicle and road is shown in Table 1.

#### 3.2.3. Experiment Design and Data Collection

Then, twenty drivers, including twelve males and eight females with an average age of 24 years, were recruited to participate in the experiment. All drivers had valid driving licenses and certain simulated driving experience. The experiment was carried out mainly to evaluate the effectiveness, safety, and acceptability of the proposed model, and did not consider strictly controlling the driver’s age, experience, and other information; therefore, this study ignored the impact of individual driver differences on model verification. Each driver underwent tests with each warning model (traditional, time-delay, and driving without a warning model) under each driving scenario; each driver needed to complete nine valid tests in total. In addition to collecting basic driving data, in each test, an Ergoneers’ driver sight capture system (Ergoneers, Geretsried, Germany) was also used to collect eye-track data. The experiment design and data collection requirements are shown in Table 2. The data collected for each test are marked with a tick. Model 1 and model 2 refer to the traditional model and the proposed time-delay model, respectively. The baseline in the table meant normal driving without a warning model.

**Figure 9:**
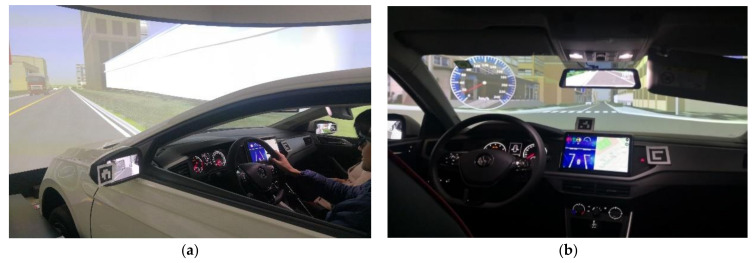
Simulation tests of V2I-based side-collision warning model for non-signalized intersections. (**a**) External environment during test. (**b**) Interior environment during test.

The total number of experiments was 60, giving a total of 180 effective tests. For each participant, to weaken the learning effect, the warning models and experiment scenarios were randomly selected to arrange each simulation. After the experiment under each warning model, participants were required to fill out a warning system evaluation scale and perform the next experiment after a five-minute rest. Photos from a typical experiment are shown in Figure 9. 

## 4. Effect Analysis of Warning Model

### 4.1. Objective Analysis of Warning Effect

#### 4.1.1. Statistical Analysis of Warning Effect

Based on the experimental data from twenty participants, a total of 110 experimental records with a warning model and 47 experimental records without a warning model were extracted for the statistical calculation of the warning effect, while the remaining 23 experimental records were considered as non-conflict. The statistical indicators are described as follows:

(1). “Available experiments” refers to the records of collision risk and warning indications that actually existed during driving. Among them, for the baseline (no warning model), according to the driving data of HV and RV within the intersection range, the commonly used surrogate safety measure TTC was used to divide the experiment fragments with collision risk and warning requirements.
(2). “Effective warning” represents the experimental fragment in which the driver immediately took avoidance measures and stopped safely after the collision warning was triggered;
(3). “Failure warning” means that the driver took risk avoidance measures before the warning was triggered;
(4). “Invalid warning” means that the system triggered the warning, but the driver did not take corresponding measures to avoid danger;
(5). “Risk resolution” means that the driver identified potential risks and took avoidance measures in advance;
(6). “Collisions” refers to vehicle collisions caused by the driver’s failure to avoid danger.

Each driver participated in each experiment for a short time (average seven minutes), and the experiment was not disturbed by other external factors; therefore, the situation wherein the driver did not perform the risk avoidance operation due to unknown reasons (such as fatigue, distraction, or aggressive driving style) was not considered. At the same time, the experimental platform was an in-loop simulation system with fast data transmission and data calculation, and there was no data loss, so the problem of warning system failure was not considered. The statistical values of warning effects are listed in Table 3.

**Figure 10:**
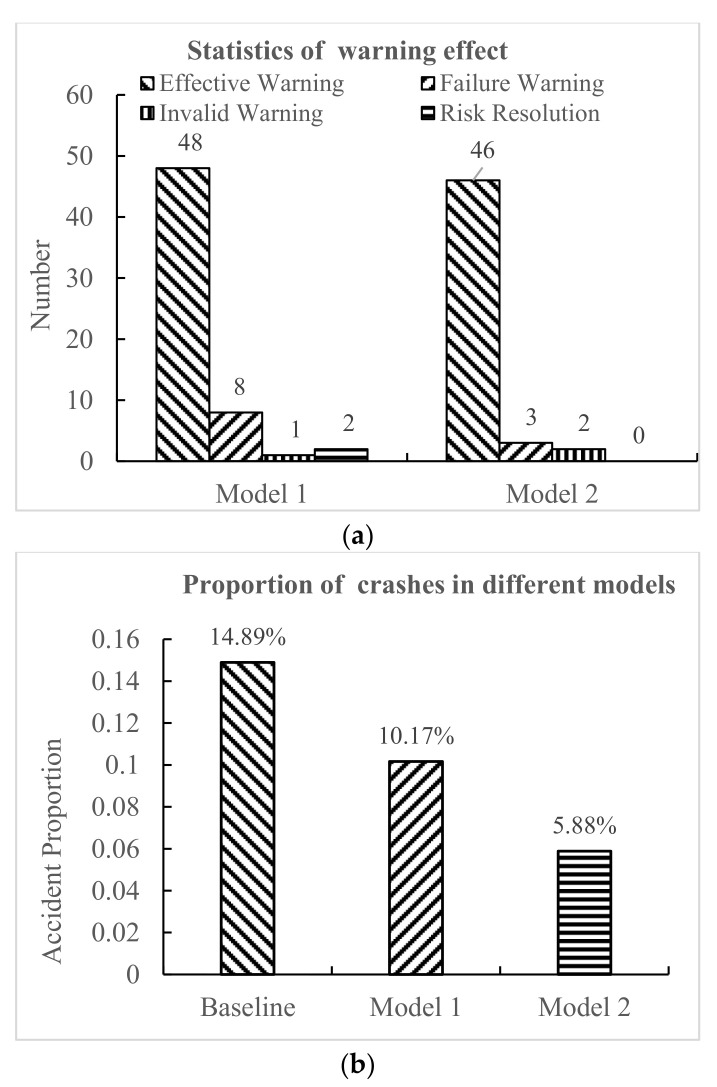
Statistics of vehicle collisions at non-signalized intersections. (**a**) Statistical values of warning. (**b**) Statistics of collision accidents.

According to the statistical value of correlation quantity of warning effect, the frequency histograms of effective warning, failure warning, invalid warning, and risk resolution under the two warning models are shown in Figure 10a. Without considering the driver’s initiative to eliminate risks, the effective warning rate of model 1 was 84.2%, while the effective warning rate of model 2 was 90.2%. As evidenced, compared with model 1, model 2 gave more effective warnings and a lower failure and invalid warning rate. Therefore, based on the results of a certain number of verification experiments, and from the perspective of the warning performance of the side-collision warning models at non-signalized intersections, model 2 outperformed model 1.

As shown in Figure 10b, the statistical proportions of vehicle collisions under baseline (i.e., without a warning model), model 1, and model 2, were 14.89%, 10.17%, and 5.88%, respectively. Therefore, relative to driving without a warning model, the use of a V2I-based intersection collision warning system reduced the rate of collisions. It shows that the existence of an intersection collision warning system is of great significance to improving driving safety. At the same time, in terms of ensuring safe passage, the driver in model 2 could show more stable ability to manipulate the vehicle and reduce the occurrence of accidents. From the safety point of view, compared to model 1, model 2 had a more prominent effect on reducing collision accidents at non-signalized intersections.

Compared to non-signalized intersections in the real world, the number of collisions at the simulated non-signalized intersections was higher during the experiment. The main reason for this was that the risk infusion method was used in simulation to constantly trigger a high-risk scenario for drivers; secondly, it may be that drivers failed to participate in the simulation with a real-world driving mentality, so the collision rate was higher.

#### 4.1.2. Analysis of Safety Indicator

(1). Analysis of warning response time

The warning response time represents the time required for the driver to take risk avoidance measures after receiving the warning information, and it reflects the speed of the driver’s response to the warning model. The statistical results of warning response times are listed in Table 4.

**Figure 11:**
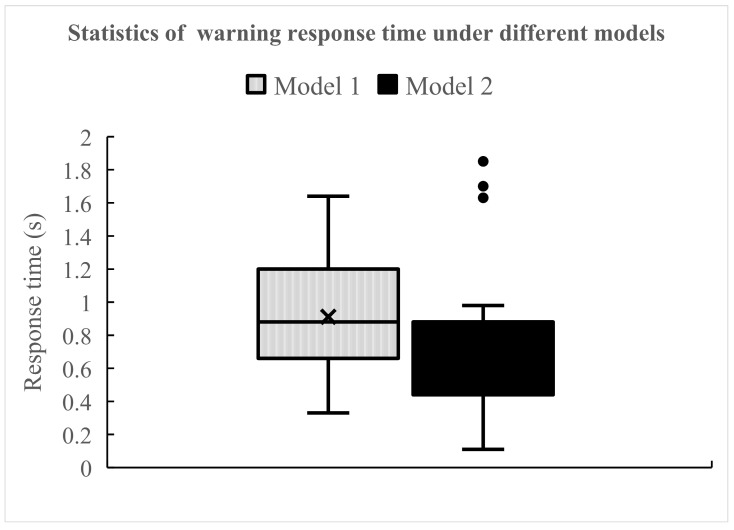
Drivers’ warning response time under different models.

As shown in Figure 11, after the warning information was triggered, the average response time of drivers was 0.91 s for model 1 and 0.78 s for model 2. In contrast, drivers reacted faster to the warning of model 2. Based on analysis results, it could be that after model 1 triggered the warning, the driver had not yet felt that there was a very urgent collision possibility, so the corresponding reaction time was slightly longer; in model 2, the driver thought that the time of warning accorded with his cognition of the potential collision possibility, so the response time was slightly shorter than that of model 1. This also shows that under the premise of ensuring safety to the greatest extent, model 1 gives drivers more time to think, make decisions, and implement risk avoidance measures. Model 2 has less operating time reserved for drivers. This may enhance the driver’s perception of potential risks, and can urge the driver to focus more quickly on possible situations, but it may also bring some pressure to the driver.

(2). Analysis of conflict time difference

The conflict time difference was selected as an index to measure the potential side-collision risk of vehicles. The conflict time difference represents the time interval between the two conflicting vehicles reaching the collision area when the avoidance measures were taken. The conflict time difference under model 1 (the time-delay model), model 2 (the traditional model), and the baseline (normal driving without warning model) was counted, and is shown in Table 5.

**Figure 12:**
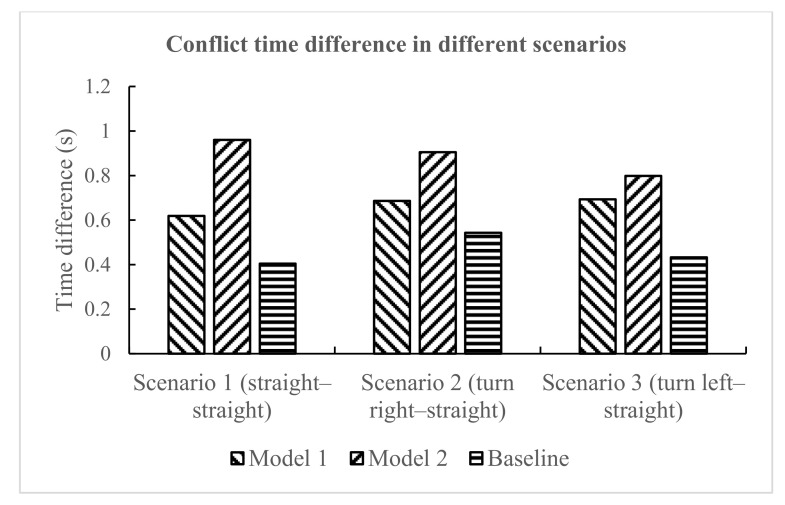
Conflict time difference in different scenarios.

The conflict time difference in different scenarios is presented in Figure 12. Regarding the warning model, whether in scenario 1, scenario 2, or scenario 3, the conflict time difference at the time of risk avoidance under model 2 was the largest, followed by model 1, and the conflict time difference under the baseline was the smallest. It shows that, compared with the no warning model, the use of the warning model had a positive effect on the driver, and reduced the possibility of collision between conflicting vehicles. At the same time, it also shows that model 2 was better than model 1 in improving driving safety performance. For the experiment scenario, the conflict time difference under different models was not obvious, and there was no specific trend.

#### 4.1.3. Analysis of Gaze Change

In classic visual-word paradigm research, language stimulation and visual context are usually combined across channels. Through eye-tracking technology, the subject’s eye movement behavior during oral comprehension is recorded to derive the subject’s understanding and reaction process [35,36]. The general process is as follows: the subjects are given pictures or texts, and at the same time they are presented instructions in an auditory manner, and the subjects are required to perform corresponding actions in accordance with the instructions. Meanwhile, the eye movement data recorded by the eye tracker are used to analyze the subject’s gaze on the target [37]. Therefore, this research was based on the visual-word paradigm; using data recorded by the eye tracker worn by participants during the experiment, the gaze changes of each participant after the system triggered the warning were extracted using the method of establishing task segments. Because the HMI used in the experiment fed back warning information to the driver through sound and pictures, and the warning form did not differ depending on the warning model, only the impact of the warning system on drivers will be discussed here.

**Figure 13:**
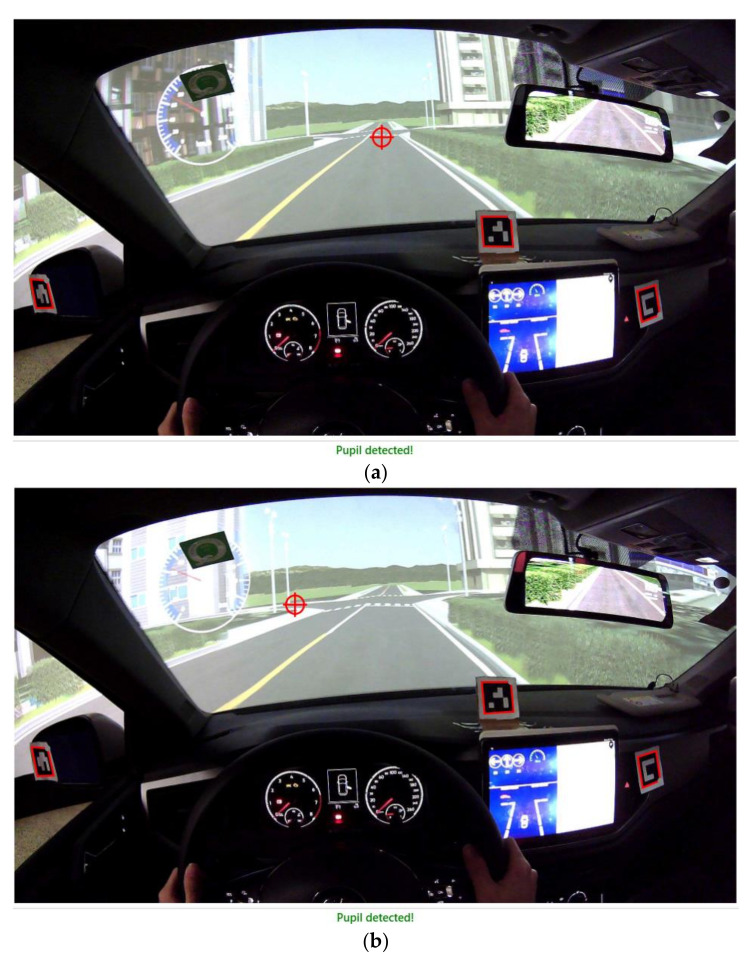
Example of driver’s gaze change processing. (**a**) When the warning was triggered, the driver’s gaze was straight ahead. (**b**) After the warning was triggered, the driver’s gaze focused on the front left.

Before the start of the experiment, the calibration and correction of the driver’s gaze point were completed by using supporting software and markers (attached to the center console inside the vehicle, and at the left and right rearview mirrors), combined with the real-time forward video of the eye tracker. The software visualized the driver’s eye movement information and front video information, and the marker was a reference coordinate system. After the calibration and correction of the eye tracker, the markers were selected by a red square, and the driver’s point of gaze automatically appeared in the video in the form of a red cross-circle, so that the driver’s gaze was always captured throughout the experiment. Therefore, when a certain time was selected, the driver’s gaze change could be judged by observing whether the position of the red cross-circle shifted before and after the time. For this study, starting from the time when the HMI triggered the warning, by observing the driver’s gaze within 250 ms after the warning (sound and icon) [38], a default 100 ms was used as a gaze process to establish task segments. Finally, according to each task segment, the driver’s gaze change information after the warning was quickly extracted. As shown in Figure 13a,b, after the warning was triggered, the driver’s gaze turned from straight ahead to left front. In the experiment, the default sampling frequency of the eye tracker was 60 Hz. 

Through the statistics of the experimental data records under the two collision warning models, it was found that there were 108 warning records and 12 no warning records. Based on the eye-tracking data, the 16 frames (about 250 milliseconds) of gaze data in the critical period of the warning prompt segment were marked and sorted, and the statistical results of the gaze changes after the warning are shown in Table 6. For experiments with warning models, “effective gaze change” means that the driver’s gaze switched from the road ahead to the direction and position indicated by the warning information; “irrelevant gaze change” means that the driver did not comply with the warning, and his gaze switched from the road ahead to an irrelevant direction and position; “failed gaze change” means that the driver’s gaze did not change at all with the direction and position indicated by the warning information. In addition, according to the available experimental segments of the baseline in Table 3, the driving data of the HV and RV were used to find out the starting time point of collision risk, and the statistical results of drivers’ gaze changes were sorted out in the same way. For the baseline (no warning model), the gaze changes were for the time period after the starting time point of collision risk. “Effective gaze change” refers to driver’s gaze switching from the road ahead to the direction and position of the dangerous target; “irrelevant gaze change” refers to the driver’s gaze switching from the road ahead to an irrelevant direction and position; “failed gaze change” refers to the driver’s gaze not changing at all. The statistics of gaze change under the warning model and the baseline were both for the situation where the collision risk (warning trigger) does occur, and their statistical significance was consistent.

**Figure 14:**
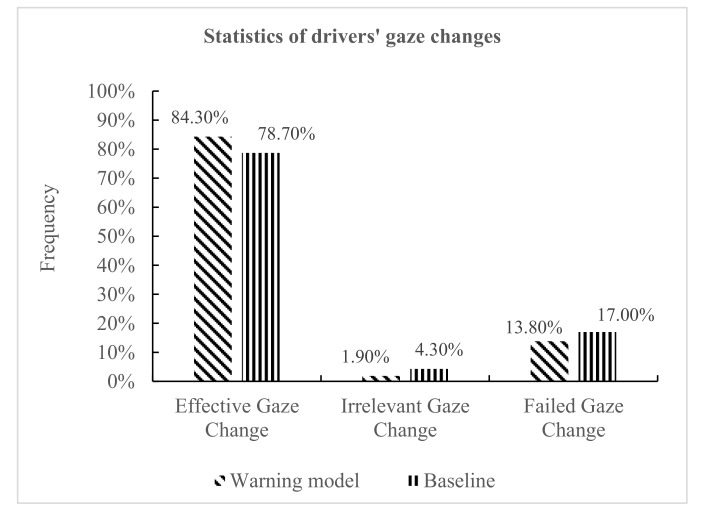
Statistics of gaze changes under warning model and no warning model scenarios.

As shown in Figure 14, the rate of effective gaze change under warning models and baseline (no warning model) was 84.3% and 78.7%, respectively; the rate of irrelevant gaze change under warning models and baseline (no warning model) was 1.9% and 4.3%, respectively; the rate of failed gaze change under warning models and baseline (no warning model) was 13.8% and 17.0%, respectively. The analysis shows that, compared with the baseline, the existence of the warning model had a significant positive impact on improving the effective gaze changes of the driver, and it urged the driver to actively perceive potential danger. When the warning was triggered, the driver could respond quickly according to the received warning information and look for potentially dangerous vehicles or dangerous areas in the field of vision. Therefore, the warning model played an important role in the driver’s perception of potential dangers and driving safety.

To analyze the impact of the scenarios on the driver’s perceived danger, the effective gaze change rates and the failed gaze change rates in different scenarios were counted separately. The statistical results of the driver’s gaze change rate in different scenarios are listed in Table 7.

**Figure 15:**
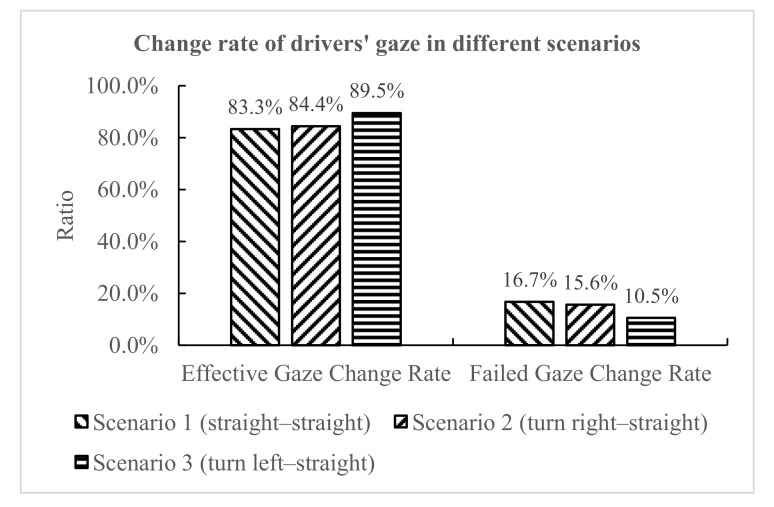
Gaze change rate in different scenarios.

As shown in Figure 15, it was found that in scenario 1, scenario 2, and scenario 3, the effective gaze change rates were 83.3%, 84.4%, and 89.5%, respectively, showing an increasing trend; the failed gaze change rates were 16.7%, 15.6%, and 10.5%, respectively, showing a decreasing trend. In scenario 3 (left turn–straight), the drivers’ gaze switched with the greatest warning information, indicating that the risk-perception demand was highest in scenario 3, and the risk-perception demand was lowest in scenario 1. In the more complex scenarios, in order to ensure the safe driving of vehicles, drivers are more willing and need to perceive potentially dangerous vehicles. Therefore, the complexity of a driving scenario had different effects on gaze change.

### 4.2. Subjective Comprehensive Evaluation of Participants

After completing the simulation experiment under one warning model, each participant filled out an evaluation of the warning system. The questionnaire adopted a 7-point Likert scale [39]. There were ten questions divided into three categories: warning system effectiveness (three questions), warning system risk (three questions), and user acceptance (four questions). By calculating the score proportion of each question and counting the comprehensive score proportion of each category, the traditional side-collision warning model and the time-delay side-collision warning model were evaluated.

**Figure 16:**
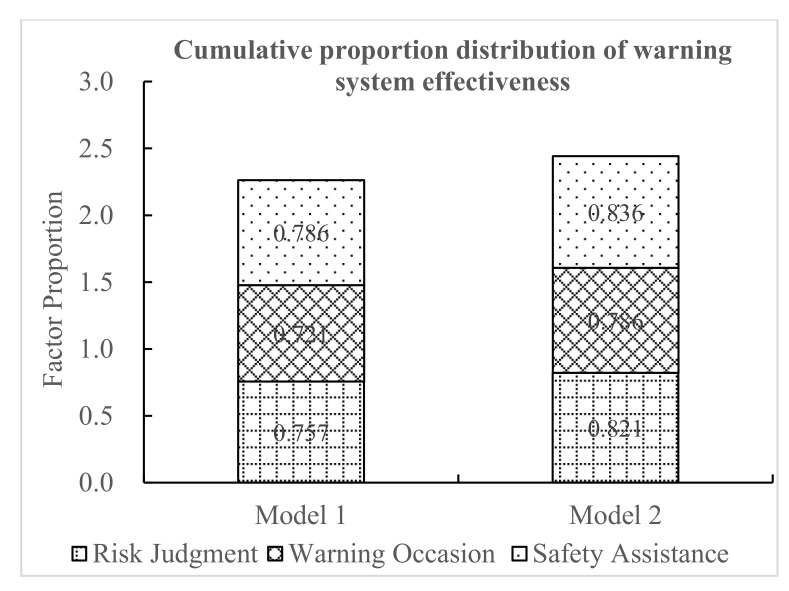
Cumulative proportion distribution of warning system effectiveness.

As shown in Figure 16, using the three questions on warning system effectiveness (risk judgment, warning occasion, and safety assistance), the cumulative score proportion of the effectiveness of the warning models was obtained. The cumulative proportion of the effectiveness of model 1 was 2.264, while the cumulative proportion of the effectiveness of model 2 was 2.443. Based on the driver’s subjective feelings, the drivers believed that the warning system of model 2 was more effective and would be more helpful to driving safety. Therefore, compared with model 1, model 2 was more effective.

**Figure 17:**
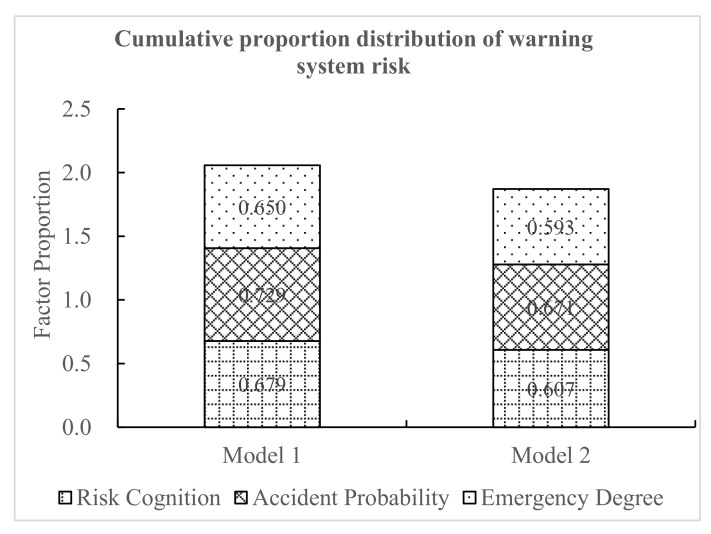
Cumulative proportion distribution of warning system risk.

As shown in Figure 17, using the three questions on warning system risk (risk cognition, accident probability, and emergency degree), the cumulative score proportion of model risk was obtained. The cumulative proportion of the risk of model 1 was 2.057, and the cumulative proportion of the risk of model 2 was 1.871. Combining the drivers’ subjective feelings, the drivers thought that the driving risk was relatively higher when the warning system of model 1 was adopted. Therefore, compared with model 2, the risk of adopting model 1 was higher. This also indirectly shows that the security of model 2 was greater.

**Figure 18:**
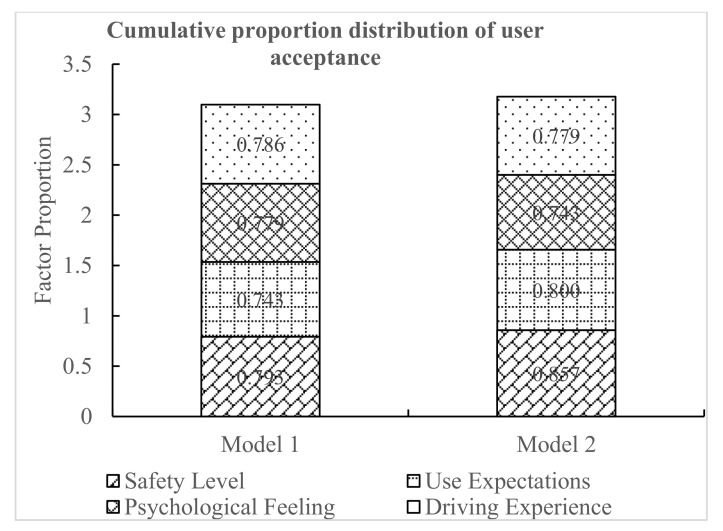
Cumulative proportion distribution of user acceptance.

As shown in Figure 18, using the four questions on user acceptance (safety level, use expectations, psychological feeling, and driving experience), the cumulative score proportion of driver adoption was obtained. The cumulative proportion of user acceptance of model 1 was 3.100, while the cumulative proportion of user acceptance of model 2 was 3.179. According to the drivers’ subjective feelings, the difference between model 1 and model 2 was relatively small, but compared to model 1, the drivers may be more willing to accept model 2.

## 5. Conclusions

Aimed at alleviating the collision risk of non-signalized intersections, this research analyzed the impact of driver response characteristics and system braking characteristics on vehicle position prediction based on a traditional side-collision warning model. A novel time-delay side-collision warning model was developed according to the motion compensation principle and *T2* risk indicator. At the same time, the simulation driving experiments were carried out by using the simulation driving platform for V2I testing, and a comparison was made between the traditional side-collision warning model and the novel time-delay side-collision model. The results showed that:

(1). The existence of a side-collision warning model at non-signalized intersections played an important role in reducing collision accident rates. Compared with the baseline (no warning model), the traditional model reduced the collision rate to 10.17%; the time-delay model reduced the collision rate to 5.88%. In terms of warning response time, the time-delay model left the driver with a shorter reaction time and urged the driver to take the risk avoidance measures faster; the conflict time difference at the risk avoidance time indicated that the time-delay model more effectively mitigated potential collision risks. According to the statistical results of drivers’ gaze changes after the warning trigger (risk generation), it could be seen that compared with the baseline (no warning model), the warning models enhanced the drivers’ perception of potential danger. It was also found that drivers had the greatest risk perception needs in scenario 3 (turn left–straight).
(2). Based on subjective evaluations completed by the drivers, statistical scores were given in terms of effectiveness, risk, and acceptance. The cumulative scores of the traditional model were 2.264, 2.057, and 3.100, respectively, while the cumulative scores of the time-delay model were 2.443, 1.871, and 3.179, respectively. Through this comprehensive analysis, it was found that drivers had a higher degree of recognition and acceptance of the time-delay warning model.

In this paper, a time-delay side-collision warning model for non-signalized intersections was developed. It improved collision warning in a complex environment and could provide critical reference for micro safety applications in V2I. However, due to the consideration of the actual cost, the intersection types and verification scenarios selected in this study were slightly deficient, so future experiments and demonstration analyses need to be carried out under more comprehensive conditions. In addition, because the remote vehicle was generated and operated by the simulation software, the model consolidated the motion constraints of the remote vehicle in the process of approaching the intersection, so the time-delay model identified risks under the assumption that the speed of the remote vehicle was constant. Therefore, subsequent research should consider the motion characteristics of the remote vehicle and establish a more detailed risk identification and side-collision warning model.

## Figures and Tables

**Table 1:** Types of interaction information based on V2I.

Information	Definition	Unit	Category
ID	The unique number of the vehicle, which is used to distinguish different vehicles.	—	Target information
Speed	The real-time speed of the vehicle as it approaches the intersection.	km/h	
Acceleration	The real-time acceleration of the vehicle as it approaches the intersection.	m/s^2^	
Coordinate position	Real-time 3D coordinate information of the vehicle.	m	
Remaining distance	The distance between the vehicle and the intersection.	m	
Outline	Length and width information of the vehicle.	m	
Special point	The center point coordinates of the intersection area.	m	Road information
Number of lanes	Number of one-way lanes at the intersection.	lane	
Lane width	Lane width in the intersection area.	m	

**Table 2:** Arrangement of experiment design and data collection.

Test Number	Scenario Type	Warning Model	Driving Data	Eye-Track Data	Evaluation Scale	Participants
1	Scenario 1	Model 1	✓	✓	✓	20 drivers
2		Model 2	✓	✓	✓	
3		Baseline	✓	✓	—	
4	Scenario 2	Model 1	✓	✓	✓	
5		Model 2	✓	✓	✓	
6		Baseline	✓	✓	—	
7	Scenario 3	Model 1	✓	✓	✓	
8		Model 2	✓	✓	✓	
9		Baseline	✓	✓	—	

**Table 3:** Statistical values of warning effects.

Statistical Indicators	Warning Model		Baseline
	Model 1	Model 2	
Available experiments	59	51	47
Effective warning	48	46	—
Failure warning	8	3	—
Invalid warning	1	2	—
Risk resolution	2	0	—
Collisions	6	3	7

**Table 4:** Statistics of warning response times.

Warning Model	Mean	Variance	Median Value	75% Quantile	90% Quantile
Model 1(s)	0.91	0.10	0.88	1.2	1.31
Model 2(s)	0.78	0.21	0.66	0.88	1.64

**Table 5:** The statistics of conflict time differences at risk aversion times.

Warning Model	Experiment Scenario		
	Scenario 1	Scenario 2	Scenario 3
Model 1 (s)	0.619	0.686	0.693
Model 2 (s)	0.961	0.905	0.799
Baseline (s)	0.404	0.543	0.433

**Table 6:** Statistics of gaze change.

Model	Gaze Statistics	Effective Gaze Change	Irrelevant Gaze Change	Failed Gaze Change	Total
Warning model	Frequency	91	2	15	108
	Proportion	84.3%	1.9%	13.8%	100%
Baseline	Frequency	37	2	8	47
	Proportion	78.7%	4.3%	17.0%	100%

**Table 7:** The proportion of gaze changes in different scenarios.

Statistics Indicators	Scenario Category		
	Scenario 1 (Straight–Straight)	Scenario 2 (Turn Right–Straight)	Scenario 3 (Turn Left–Straight)
Effective gaze change rate	83.3%	84.4%	89.5%
Failed gaze change rate	16.7%	15.6%	10.5%

## Data Availability

Data available on request due to restrictions eg privacy or ethical. The data presented in this study are available on request from the corresponding author. The data are not publicly available due to the strict management of various data and technical resources within the research team.
